# Silencing Antibiotic Resistance with Antisense Oligonucleotides

**DOI:** 10.3390/biomedicines9040416

**Published:** 2021-04-12

**Authors:** Saumya Jani, Maria Soledad Ramirez, Marcelo E. Tolmasky

**Affiliations:** Department of Biological Science and Center for Applied Biotechnology Studies, California State University Fullerton, Fullerton, CA 92831, USA; saumyajani@csu.fullerton.edu (S.J.); msramirez@fullerton.edu (M.S.R.)

**Keywords:** antisense, antibiotic resistance, RNase P, RNase H, nucleotide analogs

## Abstract

Antisense technologies consist of the utilization of oligonucleotides or oligonucleotide analogs to interfere with undesirable biological processes, commonly through inhibition of expression of selected genes. This field holds a lot of promise for the treatment of a very diverse group of diseases including viral and bacterial infections, genetic disorders, and cancer. To date, drugs approved for utilization in clinics or in clinical trials target diseases other than bacterial infections. Although several groups and companies are working on different strategies, the application of antisense technologies to prokaryotes still lags with respect to those that target other human diseases. In those cases where the focus is on bacterial pathogens, a subset of the research is dedicated to produce antisense compounds that silence or reduce expression of antibiotic resistance genes. Therefore, these compounds will be adjuvants administered with the antibiotic to which they reduce resistance levels. A varied group of oligonucleotide analogs like phosphorothioate or phosphorodiamidate morpholino residues, as well as peptide nucleic acids, locked nucleic acids and bridge nucleic acids, the latter two in gapmer configuration, have been utilized to reduce resistance levels. The major mechanisms of inhibition include eliciting cleavage of the target mRNA by the host’s RNase H or RNase P, and steric hindrance. The different approaches targeting resistance to β-lactams include carbapenems, aminoglycosides, chloramphenicol, macrolides, and fluoroquinolones. The purpose of this short review is to summarize the attempts to develop antisense compounds that inhibit expression of resistance to antibiotics.

## 1. Introduction

It has now been over 40 years since the pioneering research published by Zamenick and Stephenson, where the authors showed that the addition of a 13-mer oligonucleotide complementary to the repeated sequences located at the ends of the genome inhibited the replication of the Rous sarcoma virus in infected chicken embryo fibroblast cells [1]. These experiments were the origin of the antisense technologies, i.e., the utilization of oligonucleotides or oligonucleotide analogs to interfere with undesirable biological processes, commonly (but not exclusively), through inhibition of expression of selected genes. Since these pioneering experiments took place, a wide variety of strategies were designed to achieve therapeutic effects for the treatment of diverse diseases like viral and bacterial infections, genetic disorders, or cancer. Challenging problems such as toxicity, non-specific effects, or inability to penetrate the target were evident during the early years of antisense research, and progress was slow. However, steady progress was made, and in 1998, the Food and Drug Administration (FDA) approved fomivirsen (Vitravene^TM^), an antisense drug indicated for cytomegalovirus retinitis treatment [2]. Following this breakthrough, many antisense drugs of a diverse chemical nature and that work through different mechanisms, including the utilization of siRNAs, were approved by the Food and Drug Administration (FDA) or European Medicines Agency (EMA) (see examples in Table 1, adapted with data from references [3,4,5]) and numerous others are at different stages of development such as miravirsen, a mixmer consisting of locked nucleic acid and phosphorothioate residues for the treatment of hepatitis C [3,4,5,6,7,8,9,10,11,12].

A quick revision of the drugs approved for utilization in clinics and those in clinical trials shows that most compounds being evaluated do not target bacterial pathogens, and although several groups and companies are working on different strategies [8,24,25,26,27], the application of antisense technologies to prokaryotes still lags with respect to those that target other human diseases.

There is a variety of antisense mechanisms of inhibition exploited to design therapies for bacterial infections [8,9,24,26,28,29,30,31]. In most of these attempts, the antisense compounds target essential genes such that reducing their expression leads to bacterial death or weakening [8,9,24,26,28,30,31]. A less common strategy consists of designing antisense compounds that inhibit expression of resistance genes. Therefore, in combination with the appropriate antibiotic, they would act as adjuvants facilitating a successful treatment. The purpose of this short review is to summarize the attempts to develop antisense compounds that inhibit expression of resistance to antibiotics.

**Figure 1:**
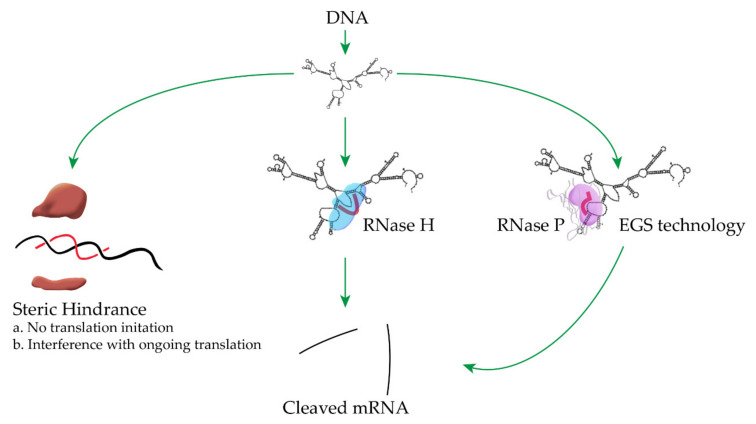
Representative mechanisms of inhibition of gene expression by antisense oligonucleotides. Left—the antisense compound binds the mRNA at the translation initiation region, preventing initiation of translation or at any location of the mRNA, in which case it will interfere with the progress of the ribosome. The figure shows only the binding at the translation initiation region. Center—an antisense oligodeoxynucleotide or analog compatible with activation of RNase H upon binding to RNA binds a single-stranded region of the target mRNA, which becomes a substrate for cleavage by RNase H. Right—External Guide Sequence (EGS) technology, an antisense oligodeoxynucleotide or analog compatible with activation of RNase P binds a single-stranded region of the target mRNA and forms the structure required to become a substrate of RNase P. Figures that include processes shown in this one can be found in recent reviews [5,9,24].

The most common mechanisms of antisense oligonucleotide inhibition of gene expression in bacteria are through degradation of the target mRNA by eliciting endogenous RNases like RNase H or RNase P, or interference with transcription or translation by steric hindrance (Figure 1) [5,9,24,32]. Thorough descriptions of different antisense mechanisms of inhibition of gene expression can be found in several excellent recent reviews [5,9,24,25,33,34,35]. In this article, we briefly summarize representative examples of the utilization of antisense strategies to counter antibiotic resistance. Although we subdivided the article in sections based on antisense mechanisms, they should be taken with caution because in numerous cases they have not been confirmed.

## 2. External Guide Sequence (EGS) Technology

External Guide Sequence (EGS) technology was intensely researched to design antisense molecules to override resistance. The general mechanism of antisense action in this case is of the type of target cleavage. EGS technology takes advantage of the bacterial host’s RNAse P. This is a ribozyme present in all organisms that plays many functions, but it was first identified by its catalytic activity that mediates digestion of the immature tRNAs 5′-end termini leading to formation of the mature tRNAs [36,37]. The RNA moiety of the enzyme is the catalytic subunit, and, in bacteria, there is usually one cofactor protein [37,38,39]. The holoenzyme recognizes structural properties in specific regions of the immature tRNA to catalyze the endonucleolytic cleavage [40,41,42].

A breakthrough for the utilization of RNase P as the basis for an antisense strategy occurred after the finding that most of the immature tRNA molecules could be removed without losing the ability to serve as substrates as long as the key regions and structure are preserved. Furthermore, bimolecular complexes with the appropriate structure, independently of the nucleotide sequence, were also substrates for RNase P [40,41,42]. These characteristics led to the idea that an oligonucleotide complementary to a target mRNA, provided that after interacting the resulting structure was appropriate, could recruit RNase P to cleave the latter, reducing the level of expression of the cognate gene [29,40,43,44]. Oligonucleotides with these properties are known as external guide sequences (EGSs).

The earliest work that showed the possibility that EGSs could be used as adjuvants to obliterate resistance followed the proof-of-concept experiments in which selected EGSs reduced expression of β-galactosidase and alkaline phosphatase in *Escherichia coli* [45]. The sequences of EGSs complementary to *bla*_TEM_ (β-lactamase) and *cat* (chloramphenicol acetyl transferase) were cloned as part of a DNA fragment consisting of a T7 promoter, the EGS sequence, a core hammerhead sequence, and a T7 terminator (T7p-EGS-HH-T7t). *E. coli* BL21(DE3) cells carrying these recombinant clones express an RNA fragment containing the EGS and the core hammerhead ribozyme, which directs a self-endonucleolytic cleavage that releases the EGS into the cytosol [46]. Addition of isopropyl β-d-1-thiogalactopyranoside to the cultures of cell harboring *bla*_TEM_ or *cat* resulted in a significant reduction of resistance to ampicillin or chloramphenicol [46]. A few years later, Gao et al. confirmed the results of conversion to susceptibility to chloramphenicol in four *E. coli* strains harboring *cat* [47].

**Figure 2:**
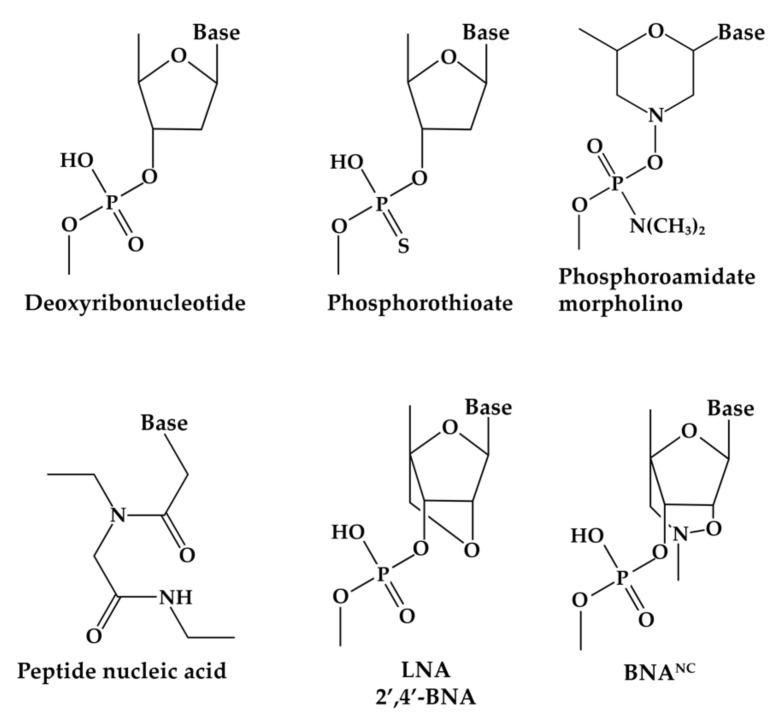
Chemical structures of nucleotide analogs mentioned in the text. The structures shown in this figure were published in the recent article by Soler Bistué et al. [53].

The early approach described in the previous paragraph was also applied to reduce expression of the *aac(6′)-Ib* gene, which codes for an acetyltransferase that inactivates amikacin and other clinically important aminoglycosides [48]. The *aac(6′)-Ib* mRNA was mapped using RNase H mapping and the sites selected as single stranded were used as targets for selection of several EGSs [49]. The most active EGSs were cloned to a DNA arrangement that was the same as that described above and upon introduction into *E. coli* cells harboring *aac(6′)-Ib,* the levels of resistance were reduced dramatically [49]. In parallel with these advances, it was necessary to explore the activity of antisense oligomers constructed with non-hydrolyzable analogs to prevent degradation. An update on nucleotide analogues has recently been published [50]. The EGSs active in reducing expression of *aac(6′)-Ib* were tested when they were synthesized using nuclease resistant analogs [51]. Oligomers containing locked nucleic acids (LNAs) (Figure 2) and deoxyribonucleotides in gapmer configuration acted as effective EGSs. Exogenous administration of the locked nucleic acids/deoxyribonucleotides EGSs to the permeable mutant *E. coli* AS19 strain harboring *aac(6′)-Ib* resulted in a reduction of levels of amikacin resistance [51]. Comparison of gapmers with different configurations, i.e., with different numbers of analogs at the ends and deoxyribonucleotides in the central region, showed large variations in activity. Furthermore, it was somewhat surprising that similar gapmers in which the LNAs were replaced by the related 2′-4′-bridged nucleic acid-NC residues (Figure 2) did not elicit RNase P-mediated degradation of the target mRNA [52]. Other nuclease resistant analogs were incapable of eliciting digestion of the target mRNA [51].

A forward step towards advancing beyond the proof-of-concept stage was the conjugation of EGSs to cell permeabilizer peptides (CPPs) that facilitate uptake of the oligomers by the target bacterial cells. The mechanism by which one CPP facilitates internalization into a Gram-negative bacterial cell was studied using the (KFF)_3_ peptide conjugated to a PNA oligomer. The results of these studies showed that CPP is essential for penetrating the outer membrane. Then, (KFF)_2_ or (KFF)_3_ are removed from the conjugate and the PNA oligomer naked or bound to a KFF tripeptide penetrates the inner membrane in a SmbA protein-dependent process [54,55]. Descriptions of mechanisms of internalization and uses of CPPs to transfer cargo inside different kinds of cells can be found in recent reviews [56,57,58,59].

While CPPs had been very useful for delivery of a variety of oligonucleotide analogs to diverse cells [56,57,58,59], their conjugation to LNAs was challenging due to the charged nature of these analogs and overcoming this stumbling block delayed the possibility of testing LNA-containing oligomers in cells in culture. The active LNA/deoxyribonucleotides gapmers were conjugated to the (RXR)_4_XB (R, arginine; X, 6 aminohexanoic acid; B, beta-alanine) peptide, a CPP for the delivery of oligonucleotides to bacterial cells [60,61]. When these compounds were added to cultures of *A. baumannii* strains that harbor *aac(6′)-Ib* they produced a reduction in the levels of resistance to amikacin [52,62]. Further studies using a gapmer composed of locked nucleic acids flanking deoxyribonucleotides conjugated to different CPPs showed that these compounds that behaved as active EGSs elicited cleavage of the *aac(6′)-Ib* mRNA in vitro. However, they had modest activities when tested in different bacteria (Jani et al., manuscript in preparation). Six CPPs were evaluated, (RXR)_4_XB (CPP1), GRKKRRQRRRPPQ (CPP2), (RFF)_3_RXB (CPP3), GWTLNSAGYLLGKINLKALAALAKKIL (CPP4), AAVALLPAVLLALLP (CPP5), and TRQARRNRRRRWRERQR (CPP6). The conjugates were tested on *K. pneumoniae* JHCK1 [63], *A. baumannii* A155 [64], and *E. coli* TOP10 (pNW1) [65] cultures containing amikacin. In the case of *K. pneumoniae* JHCK1 only the conjugate to CPP4 produced significant inhibition of growth (28%). *A. baumannii* A155 was inhibited by addition of CPP1 and CPP6 (34.2% and 34.6%, respectively), and *E. coli* TOP10 (pNW1) was inhibited by the addition of CPP1, CPP2, CPP5, and CPP6 (40.1%, 21.6%, 42.1%, and 37.6%, respectively).

Inhibition of the *cat* gene was also observed using EGSs conjugated to a CPP, in which the oligonucleotide moiety was constructed with phosphorodiamidate morpholino residues (Figure 2) [66]. Two EGSs conjugated to CPPs, with sequences targeting different locations of the *cat* mRNA, were tested in experiments where *E. coli* cells in rich medium containing chloramphenicol were mixed with the compounds. After four hours, the number of surviving cells was determined, and the results showed potent activity by each of the antisense compounds and a higher efficiency when both compounds were used together [66]. An EGS targeting the *bla*_TEM_ conjugated to a CPP was also effective in inhibiting resistance to ampicillin in *E. coli* cells after mixing the compound with the cells suspended in culture medium [67].

## 3. RNase H

Degradation of an mRNA target by RNase H is an important mechanism utilized to silence genes through antisense oligomers. RNase H is an enzyme present in all living organisms that is characterized by its endonucleolytic cleavage of RNA when it is in duplex with DNA [68]. Despite this section being called “RNase H” it must be clarified that most examples described in it have the potential to act through activation of this enzyme, but confirmation is lacking. Oligodeoxynucleotides or analogs compatible with the enzymatic activity are used to direct cleavage of the target mRNA. White al. [69] utilized antisense phosphorothioate oligodeoxynucleotides (Figure 2), which are known to induce RNase H degradation of RNA in duplex [70], to reduce the resistance to norfloxacin mediated by the expression of the multiple antibiotic resistance (*mar*) locus in *E. coli*. The *mar* locus includes two divergent transcriptional units, *marC* and *marRAB*, that are transcribed from a central regulatory region occupied by the *marO*, a regulatory locus [71]. An antisense phosphorothioate oligonucleotide that reduced expression of the transcriptional activator MarA was associated with the increase in susceptibility to norfloxacin. However, this work required the introduction of the antisense compounds by chemical transformation or electroporation and the mechanism of inhibition was not confirmed to be through activation of RNase H. Thus, steric hindrance remains a possibility [69]. Similar successes and limitations in the understanding of the mechanism of action were observed in work carried out to inhibit expression of the *aac(6′)-Ib* gene using oligodeoxynucleotides and internalizing them into the bacterial cells through electroporation [65].

Inhibition of multiple drug resistance was achieved by antisense inhibition of expression of the *Pseudomonas aeruginosa* MexAB-OprM efflux pump [72]. Accumulation of the components of this pump are associated with resistance to numerous antibiotics such as β-lactams, macrolides, quinolones, tetracycline, and chloramphenicol [73,74]. MexAB-OprM belongs to the resistance–nodulation–cell division (RND) superfamily, and it is one of many efflux pumps that can be harbored by *P. aeruginosa* [75]. MexAB-OprM includes MexB, an inner membrane transporter, OprM, an outer membrane protein, and MexA, which connects MexB with OprM. The structure of this pump and a model mechanism of drug efflux were recently proposed [74]. A phosphorothioate oligodeoxynucleotide antisense to the *oprM* mRNA was encapsulated in anionic liposomes as a delivery for internalization inside the bacterial cells [72]. Several clinical *P. aeruginosa* strains were tested and addition of the antisense resulted in a reduction of resistance levels to piperacillin, ciprofloxacin, levofloxacin, cefoperazone, imipenem, and amikacin in all cases. Unfortunately, the mechanism of inhibition has not yet been confirmed to be through cleavage of the target mRNA by RNase H. It is noteworthy that this work describes a novel anion liposome composition for encapsulation and delivery of oligonucleotides inside the bacterial cells. Liposomes were used scarcely in the past with these purposes, but new advances, including conjugation of the oligonucleotide molecule to the lipid, may increase their utilization in the future [76,77,78]. In particular, an oligonucleotide conjugated to a lipid moiety has recently proved effective in reducing the resistance level to ceftriaxone in *E. coli* cells harboring *bla*_CTX-M-15_ [78]. Extensive discussions and descriptions of inhibitors of efflux pumps can be found in recent reviews [27,79,80].

## 4. Steric Hindrance

Besides EGS technology, inhibition of resistance to ampicillin mediated by *bla*_TEM_ was also explored using other antisense techniques. One of the earliest attempts at inhibition of expression of *bla*_TEM_ utilized oligodeoxynucleotides covalently linked to a 9-aminoacridine derivative, which also proved capable of inhibiting the expression of *bla*_TEM_. The stability of the complexes between the complementary sequence of the oligonucleotide and the target was increased due to intercalation of the 9-aminoacridine derivative [81]. In this case, inhibition of gene expression occurs by interference with transcription initiation when the antisense compound binds to the transcribed strand in the open complex formed by *E. coli* RNA polymerase with the promoter [81]. Another approach consisted of a photoactivatable oligonucleotide-containing psoralen monoadducts. *E. coli* harboring *bla*_TEM_ became more susceptible to ampicillin after exposure to a photoactivated antisense 9-mer oligonucleotide bound to a psoralen 4′,5′-monoadduct [82]. Inhibition of expression of *bla*_TEM_ was also achieved using 15-mer peptide nucleic acid (PNA) oligomers (Figure 2) [83]. PNAs are oligodeoxynucleotide analogs in which the deoxyribose phosphodiester of DNA was replaced by a pseudo-peptide backbone. As a consequence, unlike DNA and RNA, which are negatively charged, PNA is a neutral compound [84]. PNA binds DNA and RNA with very high affinity, can be conjugated to CPPs, and has shown low toxicity [85,86]. PNA can interfere with gene expression, preventing transcription by binding to a DNA target or translation by binding to the target mRNA [87]. The pioneering work by Good and Nielsen where an antisense PNA targeting the *bla*_TEM_ gene reversed resistance to ampicillin utilized the PNA oligomers without any helper for internalization inside the cells. As a consequence, these authors carried out the assays using the permeable mutant *E. coli* AS19 [83].

Resistance to oxacillin was also reversed by the utilization of PNA antisense oligonucleotides in the methicillin-resistant *Staphylococcus aureus* and methicillin-resistant *Staphylococcus pseudintermedius* [88]. The antisense compounds were conjugated to three different CPPs, KFFKFFKFFK, MINWKLRLKNK, and YGRKKRRQRRR but no significant differences were found in reversion of resistance. Interestingly, measurement of the mRNA levels showed a low but significant reduction in cells treated with the antisense. This could have been a somewhat unexpected observation because PNA analogs are known not to induce RNase H cleavage. However, the phenotypic effect, i.e., reduction in resistance levels, was large, suggesting that the main mechanism of inhibition is mainly interference with protein translation [88,89,90].

Carbapenems are among the antibiotics of last resort for treatment of multidrug infections. Therefore, all efforts must be made to limit development and dissemination of resistance. The most common mechanism of resistance to carbapenems in the clinics is the presence of carbapenemases [91,92]. Other means by which bacteria resist these antibiotics are the action of efflux pumps, modification in expression or synthesis of new penicillin-binding proteins, and reduction or inactivation of expression of porins. In an effort to inhibit expression of the New Delhi metallo-β-lactamase (NDM-1), Sully et al. [93] designed antisense phosphorodiamidate morpholino oligomers conjugated to the CPP (RXR)_4_XB. The antisense sequence was complementary to the translation initiation region of *bla*_NDM-1_. The phosphorodiamidate morpholino are nucleotide analogs known to interfere with gene expression by steric hindrance. These analogs do not elicit RNase H degradation of the target mRNA. Addition of this compound to cultures of *E. coli* strains harboring *bla*_NDM-1_ produced inhibition of expression of the gene and susceptibility to meropenem [93].

PNA antisense compounds were also used to interfere with translation of the CmeA protein. This is the periplasmic component of the *Campylobacter jejuni* CmeABC efflux pump, which belongs to the RND superfamily. The PNA antisense was conjugated to a CPP, KFFKFFKFFK, and the inhibition of CmeA expression was confirmed by immunoblot analysis. This antisense compound, when added at 2 μM, was associated with a reduction of eight- and four-fold in the *C. jejuni* minimal inhibitory concentrations (MICs) of ciprofloxacin and erythromycin, respectively [94]. Extended descriptions of inhibitors of efflux pumps were recently published.

A recent approach to designing antisense compounds was recently reported. It consists of a novel semiautomated pipeline for the design, synthesis, and testing of antisense PNAs (Facile Accelerated Specific Therapeutic, FAST) [95]. This methodology is not only rapid, but it results in highly specific antisense sequences. The authors utilized the platform to design antisense compounds that act as antibiotics against five multidrug resistant *Enterobacteriaceae* clinical isolates and an antisense compound that modifies the action of carbapenem against a carbapenem-resistant *E. coli* strain [95,96]. In the latter case, the target genes were selected following a novel approach. The authors determined the expression profiles of an ertapenem-resistant, meropenem-susceptible *E. coli* strain cultured in the presence of one or the other antibiotic. Genes that showed significant expression levels in response to the presence of the antibiotic were selected as targets for FAST design of antisense PNA compounds, which were conjugated to a CPP for testing. Antisense-mediated inhibition of the genes *hycA* (regulatory protein in the formate hydrogenlyase system), *dsrB* (unknown function), and *bolA* (transcriptional regulator of several genes) potentiated carbapenem efficacy while inhibition of the genes *flhC* (transcriptional regulator of motility related functions) and *ygaC* (uknown function) resulted added resistance [95].

PNA oligomers conjugated to (KFF)_3_ designed to target the ribosome-binding site of *mcr-1* restores susceptibility to colistin in *E. coli* [97].

## 5. Final Remarks

Bacterial infections are a leading cause of death, compromised health, and disability worldwide [98]. Outbreaks of bacterial infection continue to occur, and the etiologic agents are commonly resistant to multiple antibiotics [99,100]. Furthermore, the increase in the number of antibiotic-resistant bacterial pathogens not only affects our ability to treat infectious diseases and impose an economic burden on the health system, but also complicates medical procedures that depend on prevention of infection such as surgery, treatment of cancer and other chronic diseases, organ transplants, dental work, and care for premature infants [101,102,103,104]. Despite the dire situation, researchers responded to the call for action by diverse health organizations. New strategies are being developed to continue to produce new therapies to keep pace with the accelerated rise of antibiotic resistance [105]. One of the options explored to generate new therapies that can overcome multidrug resistance is utilizing diverse antisense oligonucleotides. This technologies’ versatility permits us to envision the generation of antisense drugs that either have antibiotic activity or that disable resistance to certain antimicrobials and act as adjuvants. The high diversity of the chemistries of the nucleotide analogs used to synthesize the complementary oligonucleotide in conjunction with the different modes of action and the variety of internalization methods offer a field full of possibilities for these compounds to join the armamentarium to fight multidrug resistance. We are confident that the coming years will see the first antisense adjuvant/antibiotic formulations become a reality.

## Figures and Tables

**Table 1:** Antisense medicines approved by the Food and Drug Administration (FDA) or European Medicines Agency (EMA).

Drug	Chemistry	Route	Target	Indication	Year (FDA Approval)	Year (EMA Approval)	Company, Reference
Fomivirsen (Vitravene^TM^)	Phosphorothioate	Intravitreal	Cytomegalovirus mRNA	Cytomegalovirus infection	1998	-	Ionis Pharmaceuticals [2]
Mipomersen (Kynamro^TM^)	2′-O-Methoxyethyl, Phosphorothioate, 5-methyl cytosine	Subcutaneous	Apo-B-100 mRNA	Homozygous familial hypercholesterolemia	2013	-	Genzyme [13]
Nusinersen (Spinraza^®^)	2′-O-Methoxyethyl, Phosphorothioate, 5-methyl cytosine	Intrathecal	Pre-mRNA	Spinal muscular atrophy	2016	2017	Biogen [14]
Patisiran (Onpattro^®^)	siRNA	Intravenous	Transthyretin mRNA	hereditary transthyretin-mediated amyloidosis	2018	2018	Alnylam [15]
Inotersen (Tegsedi^®^)	2′-O-Methoxyethyl, Phosphorothioate	Subcutaneous	Transthyretin mRNA	hereditary transthyretin-mediated amyloidosis	2018	2018	Ionis Pharmaceuticals [16]
Eteplirsen (Exondys 51^®^)	Phosphorodiamidate morpholino	Intravenous	Exon 51	Duchenne muscular dystrophy	2016	2018	Sarepta [17]
Golodirsen (Vyondys 53^TM^)	Phosphorodiamidate morpholino	Intravenous	Exon 53	Duchenne muscular dystrophy	2019		Sarepta [18]
Givosiran (Givlaari^®^)	siRNA	Subcutaneous	ALS1 mRNA	Acutehepaticporphyria	2019	2020	Alnylam [19]
Milasen	2′-O-Methoxyethyl, Phosphorothioate, 5-methyl cytosine	Intrathecal	Intron 6 spice acceptor cryptic site	Neuronal ceroid Lipofuscinosis 7	* 2018		Boston Children’s Hospital [20]
Vitolarsen	Phosphorodiamidate morpholino	Intravenous	Exon 53	Duchenne muscular dystrophy	2020		Nippon Shinyaku [21]
Volanesorsen (Waylivra^®^)	2′-O-Methoxyethyl, 5-methyl cytosine, 2′-deoxy	Subcutaneous injection	Apolipoprotein C3	Familial chylomicronaemia syndrome		2019	Akcea Therapeutics [22]
Casimersen (Amondys 45 ^TM^)	Phosphorodiamidate morpholino	Intravenous	Exon 45	Duchenne muscular dystrophy	2021		Sarepta Therapeutics, Inc. [23]

* is a personalized medicine developed for a single patient. Generated with data from Dhuri et al. [5] and Crooke et al. [3,4].

## Data Availability

Not applicable.
